# Structural disconnectivity in postoperative delirium: A perioperative two‐center cohort study in older patients

**DOI:** 10.1002/alz.13749

**Published:** 2024-03-07

**Authors:** Marinus Fislage, Stefan Winzeck, Rebecca Woodrow, Florian Lammers‐Lietz, Emmanuel A. Stamatakis, Marta M. Correia, Jacobus Preller, Insa Feinkohl, Jeroen Hendrikse, Tobias Pischon, Claudia D. Spies, Arjen J. C. Slooter, Georg Winterer, David K. Menon, Norman Zacharias, BioCog Consortium, Alissa Wolf, Anika Müller, Daniel Hadzidiakos, Fatima Yürek, Friedrich Borchers, Gunnar Lachmann, Kwaku Ofosu, Maria Heinrich, Rudolf Mörgeli, Jürgen Gallinat, Simone Kühn, Edwin van Dellen, Ilse Kant, Jeroen de Bresser, Simone van Montfort, Laura Moreno‐López, Daniela Melillo, Diana Boraschi, Giacomo Della Camera, Paola Italiani, Reinhard Schneider, Roland Krause, Karsten Heidtke, Peter Nürnberg, Anja Helmschrodt, Axel Böcher, Bettina Hafen, Franz Paul Armbruster, Ina Diehl, Jana Ruppert, Katarina Hartmann, Marion Kronabel, Marius Weyer, Thomas Bernd Dschietzig, Malte Pietzsch, Simon Weber, Bernd Ittermann, Ariane Fillmer

**Affiliations:** ^1^ Department of Anesthesiology and Intensive Care Medicine Charité – Universitätsmedizin Berlin corporate member of Freie Universität Berlin and Humboldt‐Universität zu Berlin Berlin Germany; ^2^ Department of Neurology National Taiwan University Hospital Taipei City Taiwan; ^3^ Department of Computing Imperial College London BioMedIA Group London UK; ^4^ University Division of Anaesthesia, Department of Medicine University of Cambridge, Addenbrooke's Hospital Cambridge UK; ^5^ Department of Clinical Neurosciences University of Cambridge; Addenbrooke's Hospital Cambridge UK; ^6^ MRC Cognition and Brain Sciences Unit, University of Cambridge Cambridge UK; ^7^ Addenbrooke's Cambridge University Hospitals NHS Foundation Trust Cambridge UK; ^8^ Faculty of Health/School of Medicine Witten/Herdecke University Witten Germany; ^9^ Max‐Delbrueck‐Center for Molecular Medicine in the Helmholtz Association (MDC), Molecular Epidemiology Research Group Berlin Germany; ^10^ Department of Radiology University Medical Center Utrecht Utrecht The Netherlands; ^11^ Charité – Universitätsmedizin Berlin, corporate member of Freie Universität Berlin and Humboldt‐Universität zu Berlin Berlin Germany; ^12^ Max‐Delbrueck‐Center for Molecular Medicine in the Helmholtz Association (MDC), Biobank Technology Platform Berlin Germany; ^13^ Berlin Institute of Health at Charité – Universitätsmedizin Berlin, Core Facility Biobank Berlin Germany; ^14^ Departments of Psychiatry and Intensive Care Medicine, and UMC Utrecht Brain Center University Medical Center Utrecht, Utrecht University Utrecht The Netherlands; ^15^ Department of Neurology UZ Brussel and Vrije Universiteit Brussel Brussels Belgium; ^16^ Pharmaimage Biomarker Solutions GmbH Berlin Germany; ^17^ Charité – Universitätsmedizin Berlin Germany; ^18^ University Medical Center Hamburg Hamburg Germany; ^19^ University Medical Center Utrecht Utrecht Netherlands; ^20^ University of Cambridge Cambridge England; ^21^ National Research Council Napoli Naples Italy; ^22^ University of Luxembourg Luxembourg Luxembourg; ^23^ ATLAS Biolabs GmBH Berlin Germany; ^24^ Immundiagnostik AG Bensheim Germany; ^25^ Cellogic GmbH Berlin Germany; ^26^ Physikalisch‐Technische Bundesanstalt Berlin Germany

**Keywords:** dementia delirium interface, diffusion kurtosis imaging, brain health, postoperative delirium, structural disconnectivity, white matter abnormalities

## Abstract

**BACKGROUND:**

Structural disconnectivity was found to precede dementia. Global white matter abnormalities might also be associated with postoperative delirium (POD).

**METHODS:**

We recruited older patients (≥65 years) without dementia that were scheduled for major surgery. Diffusion kurtosis imaging metrics were obtained preoperatively, after 3 and 12 months postoperatively. We calculated fractional anisotropy (FA), mean diffusivity (MD), mean kurtosis (MK), and free water (FW). A structured and validated delirium assessment was performed twice daily.

**RESULTS:**

Of 325 patients, 53 patients developed POD (16.3%). Preoperative global MD (standardized beta 0.27 [95% confidence interval [CI] 0.21–0.32] *p* < 0.001) was higher in patients with POD. Preoperative global MK (−0.07 [95% CI −0.11 to (−0.04)] *p* < 0.001) and FA (0.07 [95% CI −0.10 to (−0.04)] *p* < 0.001) were lower. When correcting for baseline diffusion, postoperative MD was lower after 3 months (0.05 [95% CI −0.08 to (−0.03)] *p* < 0.001; *n* = 183) and higher after 12 months (0.28 [95% CI 0.20–0.35] *p* < 0.001; *n* = 45) among patients with POD.

**DISCUSSION:**

Preoperative structural disconnectivity was associated with POD. POD might lead to white matter depletion 3 and 12 months after surgery.

## 1 INTRODUCTION

Postoperative delirium (POD) is a highly prevalent complication after surgery and predominantly affects older patients.^1^ The exact pathomechanisms and neural substrates of POD are uncertain, and the subject of ongoing research,^2^ much of which involves perioperative neuroimaging. A range of imaging techniques have identified various brain structures as potential locations of pathology underpinning POD. Structural abnormalities of regions such as the cerebellum, hippocampus, thalamus, and basal forebrain seem to predispose to POD.^3^, ^4^ Furthermore, POD has been linked to altered connectivity in functional magnetic resonance imaging (fMRI) studies.^5^, ^6^, ^7^ Such abnormal connectivity of brain networks appears to not only play a role in contributing to the risk of POD, but also provide a biomarker of POD itself.^8^ We hypothesized that these fMRI findings might be caused by (or predispose to) underlying structural white matter changes.^6^

One study examined whole‐brain diffusion tensor imaging (DTI) prior to surgery.^9^ It revealed higher global mean diffusivity (MD) and radial diffusivity (RD) in patients who subsequently developed POD. Notably, this exploratory analysis was conducted post‐hoc and in a rather small sample of only 68 patients, of whom 17 had POD. In another study, longitudinal diffusion changes in periventricular, frontal, and temporal white matter were found to be associated with POD incidence and severity in 113 patients. POD was present in 25 patients. Therein, higher MD was shown to be the most robust DTI parameter associated with POD.^10^

The additional acquisition of postoperative diffusion weighted imaging (DWI) data is particularly beneficial as it allows insights into microstructural characteristics and development of POD and enables us to identify potential neurodegenerative progression after episodes of POD. These neurodegenerative changes might replicate or predispose to those preceding dementia. For example, baseline structural disconnectivity in terms of higher MD and lower FA was described to be associated with the development of dementia,^11^ and dementia has been identified as a strong risk factor for delirium.^12^ Furthermore, POD might not only worsen cognitive trajectories, but also aggravate or precipitate clinical dementia.^13^, ^14^ Hence, commonalities in neuroimaging findings between POD and dementia might help to understand potential overlaps between these two distinct but interrelated conditions.

To assess the cerebral microstructure, we calculated four diffusion kurtosis imaging (DKI) metrics: MD, FA, mean kurtosis (MK), and free water (FW). Taken together, these metrics are reflective of axonal neuronal and fiber integrity (FA, MK) as well as neurite density (MD).^4^, ^15^ They further indicate abnormally high, undirected diffusivity in case of microstructural damage (MD).^4^ Higher MD and lower FA as signs of abnormal white matter integrity were frequently associated with dementia.^16^ Extracellular FW increases with progressing neuronal atrophy and was observed to be associated with cognition.^17^ Cortical FW was found to first decrease in the early preclinical stage and to later rise during the late preclinical and symptomatic stages of Alzheimer's disease (AD).^18^ The properties of FW might help to distinguish between mild cognitive impairment and AD.^19^

We sought associations between these DKI metrics and the occurrence of POD, as a means of identifying global and local microstructural abnormalities that predispose to POD. To detect postoperative and longitudinal microstructural abnormalities, we undertook an analysis at a follow‐up 3 and 12 months after surgery by adjusting for preoperative DKI values. Our overarching hypothesis was that patients with POD will show signs of structural disconnectivity resembling those detected in early stages of dementia. We expected higher MD and FW, whereas FA and MK may be lower compared to surgical patients without POD preoperatively, 3 and 12 months after surgery.

## 2 METHODS

### 2.1 Study design

This analysis was based on the EU‐funded two‐center prospective observational cohort study “Biomarker Development for Postoperative Cognitive Impairment in the Elderly” study (BioCog, www.biocog.eu). BioCog aimed to develop molecular and neuroimaging biomarkers for postoperative cognitive impairment, such as POD.^20^ The study was approved by the local ethics committees (No. EA2/092/14 in Berlin, Germany and No. 14‐469 in Utrecht, Netherlands) and registered on clinicaltrials.gov prior to enrolment (NCT02265263). Participants were recruited from October 2014 to September 2019 across two study sites (Charité Universitätsmedizin, Berlin, Germany; University Medical Center Utrecht, Utrecht, Netherlands), and written informed consent was obtained. This paper has been written in accordance with the “Strengthening the reporting of observational studies in epidemiology” (STROBE) checklist.^21^

**FIGURE 1 alz13749-fig-0001:**
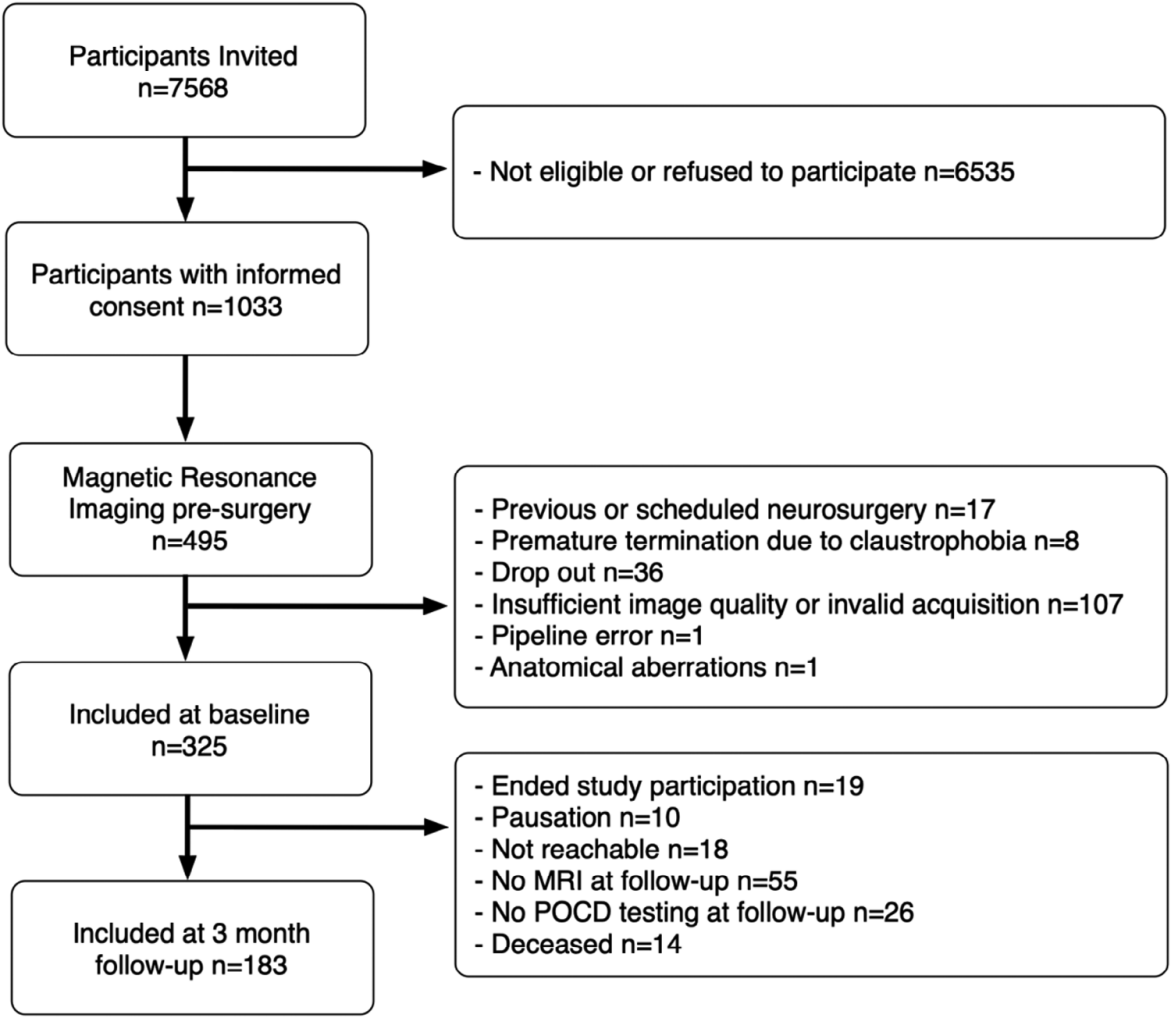
Consort diagram of participant inclusion. Flow chart detailing patients included within the wider BioCog study, and those included in this paper. Reasons for study drop out are given in the right‐hand column. Those described as “Pausation” (*n* = 10) at 3‐month follow‐up consented to later follow‐up in the study, but were not included at this time point.

The present analysis focused on older, non‐demented patients, who were scheduled for major surgery. Eligibility criteria were age over 65 years, expected surgery duration >60 min, Mini‐Mental State Examination (MMSE) score >23, and no contraindications to MRI. We further excluded participants with a history of psychiatric disease, blindness, and deafness, which could affect neurocognitive test performance. Informed consent was obtained for 1033 participants, of whom 495 had scheduled pre‐surgery MRI acquisition. Neuroimaging (MRI, DKI) from 325 participants were deemed suitable for our analysis after imaging exclusions shown in Figure 1; 39 patients from Utrecht and 286 from Berlin, to form the final study cohort.

### 2.2 Outcome assessments

POD assessment was performed in accordance with the 5th edition of the Statistical Manual of Mental Disorders (DSM‐5). This was evaluated once preoperatively and twice a day postoperatively for a maximum of 7 days or until discharge. Four validated tests were employed^22^: Nursing Delirium Screening Scale (Nu‐DESC) and Confusion Assessment Method (CAM) in non‐ICU patients, Confusion Assessment Method for the Intensive Care Unit (CAM‐ICU) in ICU patients, and additional chart review. All tests were conducted by pre‐trained study physicians, or nurses and assistants working under the supervision of a study physician. The training was designed in the format of blended learning and from bench‐to‐bedside courses.

Criteria for POD were defined during study design. Delirium was diagnosed in case of equal to or more than two cumulative points on the Nu‐DESC and/or a positive CAM score and/or a positive CAM‐ICU score and/or evidence of delirium by chart review.

**RESEARCH IN CONTEXT**

1. **Systematic review**: We reviewed the literature by using traditional databases. Human diffusion‐weighted neuroimaging studies into delirium and dementia are discussed and cited in the paper.
2. **Interpretation**: Structural disconnectivity in terms of cerebral diffusion abnormalities were shown to precede dementia. These diffusion alterations might also predispose to postoperative delirium (POD) and at the same time may be a consequence of it.
3. **Future directions**: This article highlights opportunities for future research. (1) Preoperative structural disconnectivity may help to identify patients at risk of POD. (2) Mean diffusivity should be assessed as potential predictor of POD. (3) The detrimental effects of delirium on white matter integrity could be linked to the worsening of cognitive trajectories. (4) Future studies need to investigate whether this white matter depletion is related to the onset of dementia after delirium.

### 2.3 Imaging acquisition

Whole‐brain T1‐weighted (T1w) structural MRI and diffusion‐weighted imaging were performed within 2 weeks before surgery. Acquisition protocols were harmonized as far as possible across the two imaging sites during study design and are described below.

Berlin: Participants were scanned on a 3T Siemens Trio Tim MRI scanner (Siemens Healthcare, Erlangen, Germany). T1‐weighted images were acquired with an isotropic voxel size 1.0 × 1.0 × 1.0 mm^3^, repetition time (TR) 2500 ms, and echo time (TE) 4.77 ms. DKI included one non‐diffusion weighted volume (*b* = 0 s/mm^2^) and 60 diffusion sensitized directions evenly distributed on two shells (*b* = 1000, 2500 s/mm^2^). Images were parameterized with TR = 6500 ms, TE = 100 ms, and isotropic voxel size of 2.5 × 2.5 × 2.5 mm^3^.

Utrecht: Participants were scanned on a 3T Philips Achieva scanner (Philips Healthcare, Best, The Netherlands). T1‐weighted images were acquired with an isotropic voxel size 1.0 × 1.0 × 1.0 mm^3^, TR = 7.9 ms, and TE = 4.50 ms. DKI included one non‐diffusion weighted volume (*b* = 0 s/mm^2^) and 60 diffusion sensitized directions evenly distributed on two shells (*b* = 1000, 3000 s/mm^2^). Images were parameterized with TE = 68 ms, TR = 2394 ms, and isotropic voxel size of 2.5 × 2.5 × 2.5 mm^3^.

### 2.4 Imaging preprocessing and feature extraction

**FIGURE 2:**
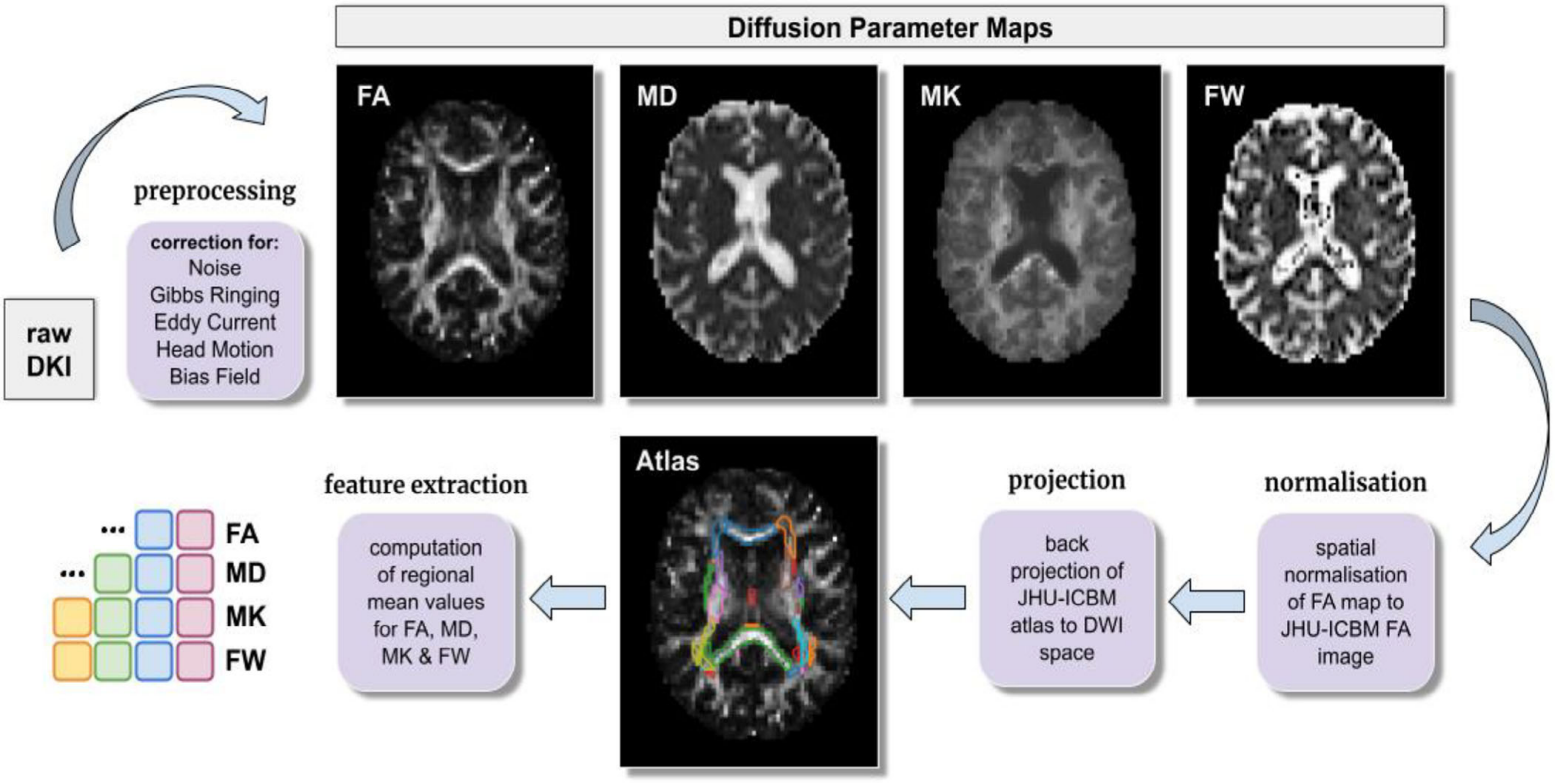
Image processing. Flow chart detailing each step of the processing of diffusion‐weighted images.

Imaging data from all participants were preprocessed with a pipeline[Fig alz13749-fig-0001] designed in‐house^23^ (Figure 2). Diffusion weighted MR images were first denoised via MPPCA,^24^ and then corrected for Gibbs ringing artifacts,^25^, ^26^ eddy current distortions as well as head motion,^27^ and lastly field inhomogeneities.^28^ Diffusion kurtosis tensors^29^ were fitted with dipy to compute fractional anisotropy (FA) and MD maps.^30^ Subsequently, FA maps were non‐linearly registered^31^ to the JHU‐ICBM atlas^32^ and transformations found were applied to backproject^31^ the JHU region atlas to patient‐specific diffusion MRI space. Thereafter, mean diffusion metrics were computed for all 48 atlas regions, providing a total of 96 image derived phenotypes.

### 2.5 Statistical analysis

We used linear mixed‐effects models to analyze association of POD with four DKI metrics (MD, FA, FW, and MK) at three time points (preoperative, 3 and 12 months) after surgery in surgical patients, resulting in twelve models. In each model, the DKI metric was treated as the dependent variable and POD as one of the independent variables of interest, as well as age, sex, baseline MMSE, comorbidity (Charlson's comorbidity index), JHU atlas region, and scanner type as covariates of no interest and a random intercept for patient. We additionally included the interaction of JHU atlas region and POD as variables to model associations of POD with regionally limited white matter alterations. The number of POD by region interactions was reduced using backward stepwise regression removing interactions using a significance criterion of *p* > 0.05. To account for multiple comparisons, we applied a conservative Bonferroni correction. The initial alpha‐level was divided by the number of regressions models, resulting in an adjusted threshold of 0.0042 (0.05/12). DKI metrics do not have SI units. Therefore, we provide the standardized beta for global DKI metrics. This also helps to compare the metrics with each other.

To model postoperative changes in white matter rather than cross‐sectional associations with POD in the data collected 3 and 12 months after surgery, we added preoperative DKI metrics as an additional independent variable into the models of DKI metrics collected at follow‐up visits.

Due to the high rate of cases that were loss‐to‐follow‐up after 12 months, we were prompted to perform a sensitivity analysis which only contained patients with DKI data for all three time points. We then compared the results to our main analyses.

During the analysis stage, two outliers were identified, whose DKI metrics abnormally differed from the rest of the cohort. This phenomenon might be due to a history of major cerebral infarction. To ensure the robustness of the results, we decided on performing a sensitivity analysis without the respective outliers.

All analyses were conducted in R v4.3.0 with additional use of the packages lme4 and lmerTest (see Supplemental R code). See the Supplemental information for the sample size calculation.

## 3 RESULTS

The final cohort in this study included 325 patients with preoperative neuroimaging; 39 from Utrecht and 286 from Berlin. These participants did not differ in age, sex, MMSE score, or delirium status from those who did not reach eligibility criteria or whose DKI data were invalid for analysis (Table S1).

Postoperatively, 53 patients developed POD during their stay in hospital (16.3%). Those with POD were significantly older than the no‐POD group (POD: median = 74.0 years [IQR 6]; no‐POD: median = 71.0 years [IQR 7]; difference −3 [confidence interval {CI} 95% −3 to (−1)] Mann–Whitney‐U: *p* = 0.003) but did not significantly differ in sex (Chi2 *p* = 0.25) (Table 1).

**TABLE 1:** Patient characteristics at baseline before surgery and information on anesthesia and surgery for the entire sample and by subgroup for those who developed postoperative delirium (POD) and for those who did not develop POD.

	All *N* = 325	No POD *N* = 272	POD *N* = 53
Age [years] – mean (SD)	72.3 (4.9)	72.0 (5.0)	73.8 (4.3)
Female sex	136 (41.8%)	110 (40.4%)	26 (49.1%)
Body mass index (BMI) – median (IQR)	26.5 (4.9) *N* = 324	26.5 (4.9) *N* = 271	26.7 (5.1)
Diabetes	71 (22%)	57 (21.0%)	14 (26.4%)
Hypertension	215 (66.2%)	179 (65.8%)	36 (67.9%)
History of stroke	25 (7.7%)	18 (6.6%)	7 (13.2%)
Malignancy	113 (34.8%)	89 (32.7%)	24 (45.3%)
Preoperative anemia	91 (28%)	74 (27.2%)	17 (32.1%)
Mini‐Mental State Examination (MMSE) – median (IQR)	29 (2)	29 (2)	28 (3)
Preoperative cognitive impairment	33 (10.2%)	24 (8.8%)	9 (17.0%)
Benzodiazepine premedication	41 (12.9%)	33 (12.1%)	8 (15.1%)
Duration of anesthesia [min] – mean (SD)	201.5 (140.2) *N* = 323	177.4 (107.4) *N* = 270	324.4 (208.8)
**Type of anesthesia**			
General	252 (77.5%)	218 (80.1%)	34 (64.2%)
Regional	18 (5.5%)	16 (5.9%)	2 (3.8%)
Combined	55 (16.9%)	38 (14.0%)	17 (32.1%)
**Type of surgery**			
Musculoskeletal	100 (30.8%)	85 (31.1%)	15 (28.8%)
Gastrointestinal	51 (15.7%)	38 (13.9%)	13 (25.0%)
Cardiovascular or thoracic	25 (7.7%)	19 (7.0%)	6 (11.5%)
Genitourinary	62 (19.1%)	50 (18.3%)	12 (23.1%)
Otorhinolaryngology	25 (7.7%)	24 (8.8%)	1 (1.9%)
Oral and maxillofacial	16 (4.9%)	15 (5.5%)	1 (1.9%)
Ophthalmology	21 (6.5%)	20 (7.3%)	1 (1.9%)
Neurosurgery	7 (2.2%)	7 (2.6%)	0 (0%)
Other	18 (5.5%)	15 (5.5%)	3 (5.8%)
**ASA score**			
ASA I	10 (3.1%)	9 (3.3%)	1 (1.9%)
ASA II	214 (65.8%)	184 (67.6%)	30 (56.6%)
ASA III	101 (31.1%)	79 (29.0%)	22 (41.5%)
Length of stay [days] – median (IQR)	6 (6)	4.5 (5)	12 (17)
Inhouse mortality	3 (0.9%)	2 (0.7%)	1 (1.9%)

*Note*: The table displays the characteristics of all patients, patients without and with delirium. For continuous variables the table states mean and standard deviation (SD) in parentheses or median and interquartile range (IQR) in parentheses. For categorical variables percentages refer to the proportion of the corresponding group (all; No Delirium; Delirium). The total N of patients with available data was added to items with cases of missing data. (ASA score ≙ American Society of Anesthesiologists’ Physical Status Classification; IQR ≙Interquartile Range ≙ 25th to 75th percentile).

### 3.1 Preoperative DKI

**FIGURE 3:**
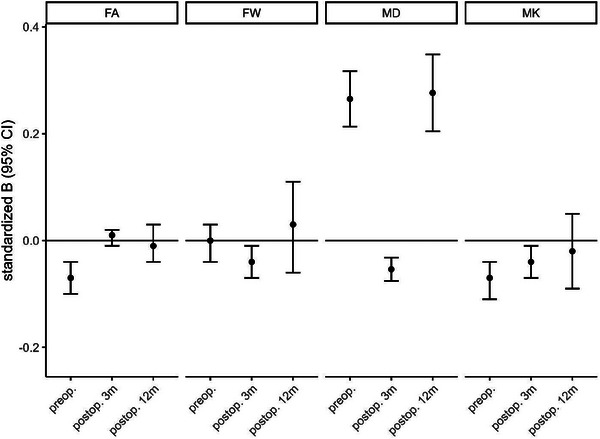
Standardized estimates. This plot displays the standardized beta (dot) and the 95% confidence interval (95% CI; error bars) for the association with postoperative delirium of four diffusion kurtosis imaging (DKI) metrics: mean diffusivity (MD), fractional anisotropy (FA), mean kurtosis (MK) and free water (FW). There are three time points for each metric: preoperative (preop.), 3 months after surgery (postop. 3m), and 12 months after surgery (postop. 12m). Covariates were age, sex, Charlson Comorbidity index, baseline Mini‐Mental State Examination, and scanner type. Baseline DKI metrics were further added to the two postsurgical analyses. Of note, after correction for multiple testing postoperative FW and MK (postop. 3m) were not significantly associated with postoperative delirium.

A total of 324 patients with complete data were analyzed. After adjustment for age, sex, baseline MMSE, CCI ≥ 1, JHU atlas region, and regional associations with POD, preoperative global MD (standardized beta 0.27 [95% CI 0.21–0.32] *p* < 0.001) was significantly higher in patients who later experienced POD, whereas both preoperative global MK (standardized beta −0.07 [95% CI −0.11 to (−0.04)] *p* < 0.001) and FA (standardized beta 0.07 [95% CI −0.10 to (−0.04)] *p* < 0.001) were lower. POD was not associated with global alterations in FW (standardized beta 0.00 [95% CI −0.04 to 0.03] *p* = 0.9) (Figure 3). Beyond global effects, several regional associations of POD with preoperative white matter alterations were observed (see Table 2): MD was particularly increased in the fornix, left superior‐frontooccipital fascicle, and left tapetum. FA was decreased in the fornix, corpus callosum, and left cingulate gyrus. MK was decreased in the left superior‐frontooccipital fascicle.

**TABLE 2 alz13749-tbl-0002:** Regional associations of POD with preoperative white matter abnormalities compared to patients without POD as indicated by interaction of JHU atlas region and POD.

Metric	Region	Hemisphere	JHU atlas number	B ± SE	*p*
MD	*Genu corpus callosum*		*3*	*0.000046* ± *0.000019*	*0.01289*
	*Body corpus callosum*		*4*	*0.000041* ± *0.000019*	*0.02955*
	***Fornix***		***6***	***0.00014*** ± ***0.000019***	**<*0.00001***
	*Sup. Cerebellar peduncle*	*R*	*13*	*0.000047* ± *0.000019*	*0.01163*
	*Ant. Corona radiata*	*R*	*24*	*0.000046* ± *0.000019*	*0.01378*
	***Fornix/Stria terminalis***	***R***	***39***	***0.000061*** ± ***0.000019***	***0.00122***
	*Sup. Frontoocc. Fsc*.	*R*	*43*	*0.000050* ± *0.000019*	*0.00797*
	***Sup. Frontoocc. Fsc***.	***L***	***44***	***0.000091*** ± ***0.000019***	**<*0.00001***
	***Tapetum***	***L***	***48***	***0.000084*** ± ***0.000019***	**<*0.00001***
FW	Genu corpus callosum		3	0.011 ± 0.005	0.03696
	Sup. Frontoocc. Fsc.	R	43	0.011 ± 0.005	0.03559
	Uncinate fsc.	L	46	−0.011 ± 0.005	0.03618
	Tapetum	L	48	−0.012 ± 0.005	0.01537
FA	***Genu corpus callosum***		***3***	−***0.016* ± *0.006***	***0.00392***
	***Fornix***		***6***	−***0.020* ± *0.006***	***0.00035***
	Post. limb of int. capsule	R	19	0.013 ± 0.006	0.01734
	***Cingulate gyrus***	***L***	***36***	−***0.016* ± *0.006***	***0.00386***
	*Fornix/stria terminalis*	*R*	*39*	−*0.013* ± *0.006*	*0.02343*
	*Fornix/stria terminalis*	*L*	*40*	−*0.013* ± *0.006*	*0.02229*
MK	*Ant. corona radiata*	*L*	*24*	−*0.023* ± *0.010*	*0.02685*
	*Superior corona radiata*	*R*	*25*	−*0.022* ± *0.010*	*0.03592*
	*Superior corona radiata*	*L*	*26*	−*0.021* ± *0.010*	*0.04917*
	Cingulum (hippocampus)	R	37	0.023 ± 0.010	0.02441
	***Sup. Frontoocc. Fsc***.	***L***	***44***	−***0.056* ± *0.010***	**<*0.00001***
	Uncinate fasciculus	*L*	*46*	−*0.026* ± *0.010*	*0.01428*

*Note*: Regions with more pronounced effects as estimated from the global association are highlighted in bold/italic. The table illustrates results for preoperative data. The number of POD by region interactions was reduced using backward stepwise regression removing interactions using a significance criterion of *p* > 0.05. Please note that the corrected alpha‐level was *p* < 0.0042. The significant regions are in bold letters.

### 3.2 Postoperative DKI at 3 months follow‐up after surgery

At the 3‐month follow‐up time point, DKI data was available for 183 patients (2 from Utrecht; 181 from Berlin). Attrition from baseline (preoperative) is summarized in Figure 1. Of 183 patients, 17 (9.3%) developed POD. The median age of patients without POD was 72 years (IQR 8) versus 72 (IQR 4) of POD patients. 70 (38.3%) were female.

After adjustment for age, sex, baseline MMSE, CCI ≥ 1, JHU atlas regions, preoperative value of the DKI metric and regionally limited associations with POD, postoperative MD (standardized beta 0.05 [95% CI −0.08 to (−0.03)] *p* < 0.001), was significantly lower in patients who had experienced POD. There was no association of POD with FA ‐(standardized beta −0.04 [95% CI −0.01 to 0.02] *p* = 0.5), FW (standardized beta 0.01 [95% CI −0.07 to (−0.01)] *p* = 0.01) or with MK (standardized beta −0.04 [95% CI −0.07 to (−0.01)] *p* = 0.01) at follow‐up after 3 months (Figure 3). Beyond global effects, several regional associations of POD with preoperative white matter alterations were observed (see Table 3): MD and FW were particularly lower in the left tapetum.

**TABLE 3 alz13749-tbl-0003:** Regional associations of POD with postoperative white matter alterations at follow‐up 3 months after surgery as indicated by interaction of JHU atlas region and POD.

Metric	Region	Hemisphere	JHU atlas number	B ± SE	*p*
MD	Fornix		*6*	−*0.000037* ± *0.000017*	*0.02850*
	***Tapetum***	***L***	***48***	−***0.000082* ± *0.000017***	**<*0.00001***
FW	***Tapetum***	***L***	***48***	−***0.020* ± *0.007***	***0.00287***
FA	Sag. stratum	R	31	−0.013 ± 0.006	0.02489
	Sup. frontoocc. Fsc.	R	43	0.015 ± 0.006	0.00932
MK	Medial lemniscus	L	*10*	−*0.031* ± *0.014*	*0.03320*
	Sup. frontoocc. Fsc.	L	*44*	−*0.028* ± *0.014*	*0.04874*

*Note*: Regions with more pronounced effects as estimated from the global association are highlighted in bold/italic. The table illustrates results for the 3 months follow‐up. The number of POD by region interactions was reduced using backward stepwise regression removing interactions using a significance criterion of *p* > 0.05. Please note that the corrected alpha‐level was *p* < 0.0042. The significant regions are in bold letters.

### 3.3 Postoperative DKI at 12 months follow‐up after surgery

The data of 45 patients were available for the 12 months follow‐up, of whom were all patients enrolled in Berlin. The median age was 72 years (IQR 9) and 26.7% (*n* = 12) were female. Among these 45 patients, there were 5 (11.1%) who were diagnosed with POD.

After adjustment for age, sex, baseline MMSE, CCI ≥ 1, JHU atlas regions, preoperative value of the DKI metric and regionally limited associations with POD, postoperative global MD (standardized beta 0.28 [95% CI 0.20–0.35] *p* < 0.001) was higher in patients who had experienced POD. POD was not associated with global FW (standardized beta 0.03 [95% CI −0.06 to 0.11] *p* = 0.5)), nor FA (standardized beta −0.01 [95% CI −0.04 to 0.03] *p* = 0.6), nor MK (standardized beta −0.02 [95% CI −0.09 to 0.05] *p* = 0.5) (Figure 3). Beyond global effects, several regional associations of POD with preoperative white matter alterations were observed (see Table 4): MD was particularly higher in the left anterior limb of the internal capsule, anterior corona radiata, and superior frontooccipital fascicle, as well as in the right fornix and stria terminalis.

**TABLE 4 alz13749-tbl-0004:** Regional associations of POD with postoperative white matter alterations at follow‐up 12 months after surgery as indicated by interaction of JHU atlas region and POD.

Metric	Region	Hemisphere	JHU atlas number	B ± SE	*p*
MD	***Ant. limb of. int. capsule***	***L***	***18***	***0.00013* ± *0.00004***	***0.00043***
	***Ant. corona radiata***	***L***	***24***	***0.00017* ± *0.00004***	**<*0.00001***
	***Fornix/stria terminalis***	***R***	***39***	***0.00011* ± *0.00004***	***0.00414***
	*Fornix/stria terminalis*	*L*	*40*	*0.00009* ± *0.00004*	*0.01570*
	***Sup. frontoocc. Fsc***.	***L***	***44***	***0.00014* ± *0.00004***	***0.00011***
FW	Fornix/stria terminalis	L	40	0.029 ± 0.017	0.03360
	Tapetum	R	47	0.029 ± 0.017	0.03754
	Tapetum	L	48	−0.037 ± 0.014	0.00724
FA	Cingulum (hippocampus)	L	38	0.024 ± 0.012	0.03692
	Tapetum	R	47	−0.023 ± 0.012	0.04590
MK	Corticospinal tract	L	8	−0.056 ± 0.026	0.03428
	Sup. Cerebellar peduncle	R	13	0.107 ± 0.026	**<*0.00001***
	Cerebral peduncle	L	16	−0.073 ± 0.027	0.00597

*Note*: Regions with more pronounced effects as estimated from the global association are highlighted in bold/italic. The table illustrates results for the 12 months follow‐up. The number of POD by region interactions was reduced using backward stepwise regression removing interactions using a significance criterion of *p* > 0.05. Please note that the corrected alpha‐level was *p* < 0.0042. The significant regions are in bold letters.

### 3.4 Sensitivity analysis of patients with available data after 12 months

We performed the same analysis for all time points with patients that had available data for the 12‐month follow‐up. As with the entire cohort, preoperative MD (standardized beta 0.10 [95% CI 0.03–0.16] *p* = 0.007) was higher and FA (standardized beta −0.17 [95% CI −0.26 to 0.08] *p* < 0.001) was lower in patients with POD. Neither preoperative FW (standardized beta 0.03 [95% CI −0.06 to 0.11] *p* = 0.5) nor MK (standardized beta −0.05 [95% CI −0.11 to 0.00] *p* = 0.07) were associated with POD.

Only 41 patients of the 45 that were present for the 12‐month follow‐up were also attending the follow‐up 3 months after surgery. Of those only 3 patients had POD. MD (standardized beta 0.09 [95% CI 0.04–0.14] *p* < 0.001) was higher in patients who had experienced POD. POD was associated with global FW (standardized beta 0.15 [95% CI −0.07 to 0.23] *p* < 0.001). FA (standardized beta 0.05 [95% CI −0.01 to 0.10] *p* = 0.11) nor MK (standardized beta −0.05 [95% CI −0.11 to 0.01] *p* = 0.11) were associated with POD after 3 months.

### 3.5 Outlier

Global associations of preoperative data remained unchanged after the exclusion of the two outliers. For the follow‐up after 3 months, global associations changed for FA (*B* = 0.01 [0.00; 0.02], *p* = 0.03) and FW (*B* = 0.00 [−0.01; 0.01], *p* = 0.6) after the removal of two outliers. No or minor changes were observed for MD (*B* = −0.00007 [−0.00010; −0.00005], *p* < 0.001) and MK (*B* = −0.02 [−0.04; 0.00], *p* = 0.061). Analyses of the data after 12 months were not affected by outliers since the two patients with major stroke did not return for follow‐up at this time point.[Table alz13749-tbl-0002], [Table alz13749-tbl-0003], [Table alz13749-tbl-0004]

## 4 DISCUSSION

DKI revealed signs of structural disconnection (lower FA and MK, higher MD) were present in patients that developed POD in comparison to patients without POD. Patients with global white matter abnormalities are more vulnerable to experience POD. Although significant interaction effects indicate more pronounced alterations are found especially in the fornix, left superior‐frontooccipital fascicle and the left tapetum, it remains unclear whether there is a regionally distinct white matter signature for delirium. Our findings suggest that POD is rather associated with a global structural disconnectivity than characteristic patterns of abnormalities in single regions. At the 3 months follow‐up after surgery, global MD was lower in patients with POD, but was higher again 12 months after surgery. None of the other postoperative DKI metrics (FA, MK, FW) turned out to be associated with POD.

FW might not be helpful in identifying brain structural correlates of POD.^4^ Although preoperative FA and MK were associated with the later occurrence of POD, they were not associated with POD after surgery. The use of FA was recently discouraged due to the impaired interpretability in regions of white matter fiber crossings.^15^ Instead, MD was recommended. Higher preoperative MD appears to be a common observation across most delirium‐related diffusion MRI studies. This applies to areas of gray and white matter alike.^3^, ^4^, ^9^ In a longitudinal study determining perioperative changes in diffusion 1 year after surgery, FA was lower and MD higher in patients with POD. However, MD was reported to be the most robust marker for a longitudinal study setting.^3^ The higher preoperative global MD of POD patients is in line with previous cross‐sectional findings, whereas lower global FA was not associated with POD in an exploratory study.^9^ This discrepancy might be caused by voxel‐based approaches in past publications, which included gray matter areas, and the post‐hoc design with a rather small sample size.

The inclusion of the preoperative DKI metrics to the postoperative models enables us to draw conclusions  about the longitudinal development of white matter abnormalities of POD patients in comparison to patients without POD. Furthermore, our study is the first to analyze diffusion‐weighted data 3 months after surgery, whereas other studies have only focused on diffusion changes after 12 months.^10^ The analysis of postoperative data after 3 months suggested paradoxically lower global MD in patients with POD. Since these alterations were observed after correction for baseline values, it seems reasonable to assume that these findings are sequelae of POD. Lower postoperative MD values in POD patients could perhaps be ascribed to pseudo‐normalizations which were reported in a longitudinal study on traumatic brain injury.^33^ This sequence of events suggests an initial increase in MD due to an acute insult which might be related to surgery. In the subacute stage, MD pseudonormalizes, but the white matter insult is now responsible for persistent white matter loss. However, to detect an acute insult that correlates with a postsurgical increase in MD, neuroimage acquisition in the early days after surgery is required. Further studies are needed to better contextualize this finding.

Although the loss to follow‐up was high at 12 months after surgery, we again observed globally increased MD in patients who had experienced delirium even after adjustment for baseline MD. The pattern of particularly altered brain regions after POD resembled the preoperatively observed pattern, comprising the left superior‐frontooccipital fascicle and the left fornix. Due to the small resulting sample size and the low remaining fraction of patients with POD at the 12 months follow‐up, the results for this time point must be cautiously interpreted. Furthermore, we would refrain from drawing conclusions about overlaps between structural disconnectivity patterns 1 year after episodes of POD and the patterns preceding dementia.^11^ However, it is worth noting that global MD was higher across both study populations.

Structural disconnectivity may represent diffuse brain damage that leads to increased vulnerability to both delirium and dementia. Furthermore, regional hypometabolism was discussed earlier as a potential link between delirium and dementia.^34^ A brain characterized by white matter abnormalities to a degree of structural disconnection might be incapable of buffering stressors such as hypometabolism as they occur during surgery induced by anesthetics.^35^ A principal contribution of this study to the literature is to increase our understanding of the consequences of delirium. We have shown that episodes of POD might be detrimental for white matter integrity. Together with the loss of gray matter volume after episodes of POD,^36^ our findings could represent the structural correlate of worsening cognitive trajectories in POD patients.^37^ Future studies need to investigate whether this white and gray matter depletion after episodes of delirium may lead to cognitive decline and eventually the development of dementia.

We further ascertained the robustness of our results by excluding two outliers that might have obfuscated our results. This is the first neuroimaging study into delirium to assess the impact of outliers. However, we recognize the need for a systematic investigation of the impact of extreme data points, especially for smaller studies. Despite our large sample size, the removal of two subjects with major stroke slightly affected our findings for FA and FW after 3 months, potentially limiting the reliability for these two modalities. However, results for MD and MK seemed relatively robust.

Taking imaging studies on delirium together, we must acknowledge that POD might be caused by variety of indicators of brain health. However, our DKI findings might help to translate imaging findings into clinical practice. In particular, MD values are easy to obtain. Preoperatively, patients at risk of POD could potentially be identified through their elevated global MD. However, whether our results can be translated into clinical practice (e.g., to predict delirium), remains a matter to be investigated by future studies.

## 5 LIMITATIONS

Our primary limitation is that only 45 patients had available DKI data for baseline and the 12 months follow‐up. Of these, only 5 patients were diagnosed with POD. The large number lost to follow‐up hampers drawing conclusions about structural disconnectivity 1 year after surgery and could be a source of bias. However, the data after 1 year might help to understand the polarity inversion of MD among patients with POD. We have conducted a sensitivity analysis only with the 45 patients who have available data for the follow‐up after 12 months. Preoperative results resemble those of the entire cohort, only MK was not significantly associated with POD anymore. Noteworthy, only 41 of the 45 patients received an MRI at the 3‐months follow‐up and of whom only three had delirium after surgery. Therefore, we are convinced that this part of the analysis does not allow for drawing conclusions. Future studies should address the issue of high loss‐to‐follow‐up rates.

We further acknowledge limitations regarding data selection. Our data were acquired across two centers in Berlin and Utrecht, which showed a strong asymmetry in collection (286 patients from Berlin, 39 patients from Utrecht). This site difference was largely due to quality control, as 107 patients from Utrecht were excluded due to image acquisition problems. This could have introduced a selection bias toward those patients who were included in the final study cohort. However, we found no significant differences in sociodemographic and clinical factors between those patients included versus those excluded. We, therefore, suggest any influence of selection bias is minimal.

Finally, there is no current standardization of anesthesiology management. Depth of anesthesia was continuously monitored using intraoperative electroencephalogram; however, we were unable to account for possible effects of intraoperative handling. Moreover, no interrater reliability assessments were performed for POD testing, which means that deviations in delirium ratings remain a possibility.

## 6 CONCLUSION

Global functional alterations were hypothesized to be an important risk factor in POD. Microstructural abnormalities as depicted by DKI may be the structural correlate. Our findings indicate that abnormal structural disconnectivity predisposes patients to POD. By correcting for preoperative diffusion metrics, we could also show that episodes of POD may be detrimental for the cerebral microstructure.

## AUTHOR CONTRIBUTIONS

credit.niso.org

Marinus Fislage: Conceptualization, data curation, formal analysis, investigation, methodology, software, validation, visualization, writing—original draft, and writing—review & editing. Stefan Winzeck: Conceptualization, data curation, formal analysis, investigation, methodology, software, validation, visualization, writing—original draft, and writing—review & editing. Rebecca Woodrow: Conceptualization, investigation, methodology, visualization, writing—original draft, and writing—review & editing. Florian Lammers‐Lietz: Conceptualization, formal analysis, investigation, visualization, writing—original draft, and writing—review & editing. Emmanuel A. Stamatakis: Conceptualization, resources, investigation, supervision, and writing—review & editing. Marta M. Correia: Conceptualization, methodology, supervision, writing—original draft, and writing—review & editing. Jacobus Preller: Conceptualization and writing—review & editing. Insa Feinkohl: Supervision and writing—review & editing. Tobias Pischon: Funding acquisition, supervision, and writing—review & editing. Jeroen Hendrikse: Supervision and writing—review & editing. Arjen J.C. Slooter: Funding acquisition, project administration, resources, and writing—review & editing. Claudia D. Spies: Funding acquisition, project administration, resources, and writing—review & editing. Georg Winterer: Funding acquisition, project administration, resources, supervision, and writing—review & editing. David M. Menon: Conceptualization, resources, supervision, and writing—review & editing. Norman Zacharias: Project administration, conceptualization, resources, supervision, and writing—review & editing.

## CONFLICT OF INTEREST STATEMENT

This publication is part of Marinus Fislage's PhD/MD degree. Author disclosures are available in the Supporting information.

## CLINICAL TRIAL NUMBER

NCT02265263 (https://clinicaltrials.gov/ct2/show/results/NCT02265263).

## CONSENT STATEMENT

All patients have given written informed consent.

## Supporting information

Supporting Information

Supporting Information

Supporting Information

Supporting Information

Supporting Information

## COLLABORATORS

The BioCog Consortium further consists of Alissa Wolf, Anika Müller, Daniel Hadzidiakos, Fatima Yürek, Friedrich Borchers, Gunnar Lachmann, Kwaku Ofosu, Maria Heinrich, Rudolf Mörgeli (*all Charité – Universitätsmedizin Berlin*), Jürgen Gallinat, Simone Kühn (*all University Medical Center Hamburg*), Edwin van Dellen, Ilse Kant, Jeroen de Bresser, Simone van Montfort (*all University Medical Center Utrecht*), Laura Moreno‐López (*University of Cambridge*), Daniela Melillo, Diana Boraschi, Giacomo Della Camera, Paola Italiani (*all National Research Council Napoli*), Reinhard Schneider, Roland Krause (*all University of Luxembourg*), Karsten Heidtke, Peter Nürnberg (*all ATLAS Biolabs GmBH, Berlin*), Anja Helmschrodt, Axel Böcher, Bettina Hafen, Franz Paul Armbruster, Ina Diehl, Jana Ruppert, Katarina Hartmann, Marion Kronabel, Marius Weyer, Thomas Bernd Dschietzig (*all Immundiagnostik AG, Bensheim*), Malte Pietzsch, Simon Weber (*all Cellogic GmbH, Berlin*), Bernd Ittermann, and Ariane Fillmer (*all Physikalisch‐Technische Bundesanstalt, Berlin*).
