# Supplementary material for: Structural disconnectivity in postoperative delirium: A perioperative two‐center cohort study in older patients
Source: Alzheimers Dement. 2024 Mar 7;20(4):2861–72. doi: 10.1002/alz.13749 (PMC11032567; doi:10.1002/alz.13749)
Supplement: Supplementary file 1 — Supporting Information [file ALZ-20-2861-s004.docx]

**Supplemental Information - Sample Size calculation**

Assuming an incidence for a perioperative neurocognitive disorder of 20-30% and a 10% drop-out rate, an effect size of Hedges G 0.5 to yield 80% power (α=5%, two-sided) roughly requires a sample size of 200 patients for any imaging biomarker.
