# Supplementary material for: Structural disconnectivity in postoperative delirium: A perioperative two‐center cohort study in older patients
Source: Alzheimers Dement. 2024 Mar 7;20(4):2861–72. doi: 10.1002/alz.13749 (PMC11032567; doi:10.1002/alz.13749)
Supplement: Supplementary file 3 — Supporting Information [file ALZ-20-2861-s003.docx]

**Supplemental Table 1 - Characteristics of included patients (Included) and of patients with invalid or missing DKI data (Excluded)**

| Variable | **Included**  **=325** | **Excluded**  **=159** |  |
| --- | --- | --- | --- |
| Age – mean (SD) | 72.31 (4.94) | 71.79 (4.82) | **P = 0.27^1^** |
| Sex – female/male | 136/189 | 56/103 | **P = 0.16^2^** |
| Mini-Mental-State-Examination (MMSE) – mean (SD) | 28.55 (1.38) | 28.57 (1.40) | **P = 0.86^1^** |
| Postoperative Delirium – number (percentage) | 53 (16.31%) | 22 (13.8%) | **P= 0.52^2^** |

**^1^** unpaired, two-tailed t-test

2 Chi^2^ test
