# Supplementary material for: Structural disconnectivity in postoperative delirium: A perioperative two‐center cohort study in older patients
Source: Alzheimers Dement. 2024 Mar 7;20(4):2861–72. doi: 10.1002/alz.13749 (PMC11032567; doi:10.1002/alz.13749)
Supplement: Supplementary file 6 [file ALZ-20-2861-s005.odt]

#-------------SET-UP ENVIRONMENT----------------------------------------

.libPaths("C:/Users/lamme/Documents/R/win-library/4.3") # load packages

library(foreign)

library(tidyr)

library(tidyverse)

library(fastDummies)

library(ggplot2)

library(corrplot)

library(lme4)

library(lmerTest)

#-------------DATA PREPARATION-----------------------------------------

dti.data="C:/Users/lamme/Documents/Paper/Publikation Fislage DTI/Data/biocog_dwi_jhu_idps.csv" # define filepath

dti.data=read.csv(dti.data, header=TRUE) # read DTI data to dataframe

dti.data=subset(dti.data, time_point!="travelling") # create dataframe without travelling heads

dti.data=subset(dti.data, category!="controls")  # create dataframe without controls (no clinical data available)

md.data= subset(dti.data, metric=="MD") # create dataframe for each modality

fw.data= subset(dti.data, metric=="FW")

fa.data= subset(dti.data, metric=="FA")

mk.data= subset(dti.data, metric=="MK")

data.columns=c("X1","X2","X3","X4","X5","X6","X7","X8","X9","X10","X11","X12","X13","X14","X15","X16","X17","X18","X19","X20","X21","X22","X23","X24","X25","X26","X27","X28","X29","X30","X31","X32","X33","X34","X35","X36","X37","X38","X39","X40","X41","X42","X43","X44","X45","X46","X47","X48") # create list of transposed variables

 

md.data.mx.pre=as.matrix(select(subset(md.data, time_point=="pre"), data.columns))                 # create

md.data.mx.post=as.matrix(select(subset(md.data, time_point=="post"), data.columns))               # matrices

md.data.mx.post12=as.matrix(select(subset(md.data, time_point=="post_oneYearFU"), data.columns))   # for

fw.data.mx.pre=as.matrix(select(subset(fw.data, time_point=="pre"), data.columns))                 # visualization

fw.data.mx.post=as.matrix(select(subset(fw.data, time_point=="post"), data.columns))               # in

fw.data.mx.post12=as.matrix(select(subset(fw.data, time_point=="post_oneYearFU"), data.columns))   # correlation

fa.data.mx.pre=as.matrix(select(subset(fa.data, time_point=="pre"), data.columns))                 # plots

fa.data.mx.post=as.matrix(select(subset(fa.data, time_point=="post"), data.columns))                

fa.data.mx.post12=as.matrix(select(subset(fa.data, time_point=="post_oneYearFU"), data.columns))

mk.data.mx.pre=as.matrix(select(subset(mk.data, time_point=="pre"), data.columns))                 

mk.data.mx.post=as.matrix(select(subset(mk.data, time_point=="post"), data.columns))                

mk.data.mx.post12=as.matrix(select(subset(mk.data, time_point=="post_oneYearFU"), data.columns))

corrplot(cor(md.data.mx.pre, use="complete.obs", method="spearman"), method="color")    # plot heatmaps for

corrplot(cor(md.data.mx.post, use="complete.obs", method="spearman"), method="color")   # between-region

corrplot(cor(md.data.mx.post12, use="complete.obs", method="spearman"), method="color") # correlations

corrplot(cor(fw.data.mx.pre, use="complete.obs", method="spearman"), method="color")

corrplot(cor(fw.data.mx.post, use="complete.obs", method="spearman"), method="color")

corrplot(cor(fw.data.mx.post12, use="complete.obs", method="spearman"), method="color")

corrplot(cor(fa.data.mx.pre, use="complete.obs", method="spearman"), method="color")

corrplot(cor(fa.data.mx.post, use="complete.obs", method="spearman"), method="color")

corrplot(cor(fa.data.mx.post12, use="complete.obs", method="spearman"), method="color")

corrplot(cor(mk.data.mx.pre, use="complete.obs", method="spearman"), method="color")

corrplot(cor(mk.data.mx.post, use="complete.obs", method="spearman"), method="color")

corrplot(cor(mk.data.mx.post12, use="complete.obs", method="spearman"), method="color")

md.data=pivot_longer(data=md.data, values_to="md", cols=data.columns, names_to="region") # transposing dataframe,

fw.data=pivot_longer(data=fw.data, values_to="fw", cols=data.columns, names_to="region") # so $region becomes 

fa.data=pivot_longer(data=fa.data, values_to="fa", cols=data.columns, names_to="region") # an independent variable 

mk.data=pivot_longer(data=mk.data, values_to="mk", cols=data.columns, names_to="region") # to adjust for

md.data=pivot_wider(data=md.data, names_from=time_point, values_from=md, names_prefix="md.") # transposing dataframe,

fw.data=pivot_wider(data=fw.data, names_from=time_point, values_from=fw, names_prefix="fw.") # so the metric variable is

fa.data=pivot_wider(data=fa.data, names_from=time_point, values_from=fa, names_prefix="fa.") # split into three variables by

mk.data=pivot_wider(data=mk.data, names_from=time_point, values_from=mk, names_prefix="mk.") # session (baseline, 3m, 12m)

demo.data="C:/Users/lamme/Documents/Paper/Publikation Fislage DTI/Data/demographics.csv" # define filepath for demographic data

demo.data=read.csv(file=demo.data, header=TRUE) # load demographic data to dataframe

demo.data=subset(demo.data, time_point!="travelling") # create dataframe without travelling heads

demo.data=subset(demo.data, category!="controls")  # create dataframe without controls (no clinical data available)

md.data=inner_join(md.data, demo.data, by="subject_id") # add the demographic data to each DTI metric dataset

fw.data=inner_join(fw.data, demo.data, by="subject_id") # the resulting dataset includes only subjects which

fa.data=inner_join(fa.data, demo.data, by="subject_id") # are included in both datasets (i.e., NAs are removed)

mk.data=inner_join(mk.data, demo.data, by="subject_id")

ggplot(data=md.data, aes(md.pre))            + geom_histogram() + facet_wrap(.~region) # plot histograms

ggplot(data=md.data, aes(md.post))           + geom_histogram() + facet_wrap(.~region)

ggplot(data=md.data, aes(md.post_oneYearFU)) + geom_histogram() + facet_wrap(.~region)

ggplot(data=fw.data, aes(fw.pre))            + geom_histogram() + facet_wrap(.~region)

ggplot(data=fw.data, aes(fw.post))           + geom_histogram() + facet_wrap(.~region)

ggplot(data=fw.data, aes(fw.post_oneYearFU)) + geom_histogram() + facet_wrap(.~region)

ggplot(data=fa.data, aes(fa.pre))            + geom_histogram() + facet_wrap(.~region)

ggplot(data=fa.data, aes(fa.post))           + geom_histogram() + facet_wrap(.~region)

ggplot(data=fa.data, aes(fa.post_oneYearFU)) + geom_histogram() + facet_wrap(.~region)

ggplot(data=mk.data, aes(mk.pre))            + geom_histogram() + facet_wrap(.~region)

ggplot(data=mk.data, aes(mk.post))           + geom_histogram() + facet_wrap(.~region)

ggplot(data=mk.data, aes(mk.post_oneYearFU)) + geom_histogram() + facet_wrap(.~region)

md.data=dummy_cols(.data=md.data, select_columns="region", remove_first_dummy=TRUE) # create dummy variables

fw.data=dummy_cols(.data=fw.data, select_columns="region", remove_first_dummy=TRUE) # for $region

fa.data=dummy_cols(.data=fa.data, select_columns="region", remove_first_dummy=TRUE)

mk.data=dummy_cols(.data=mk.data, select_columns="region", remove_first_dummy=TRUE)

reg.dummies.frm	=paste0("I(region_X",2:48,"-0.5)",collapse="+") # creates a string for all regions to use as centerered variables for interaction terms in the model formula

#-----PREPARING REGRESSION MODELS FOR PREOPERATIVE DATA ANALYSIS------

formula.md.pre.ext=as.formula(paste0("md.pre~I(POD-0.5)*(",reg.dummies.frm,")+age+sex+MMSE+cc_score_pre +(1|subject_id)")) # creates formulas

formula.fw.pre.ext=as.formula(paste0("fw.pre~I(POD-0.5)*(",reg.dummies.frm,")+age+sex+MMSE+cc_score_pre +(1|subject_id)")) # of extended models

formula.fa.pre.ext=as.formula(paste0("fa.pre~I(POD-0.5)*(",reg.dummies.frm,")+age+sex+MMSE+cc_score_pre +(1|subject_id)")) # of preoperative data

formula.mk.pre.ext=as.formula(paste0("mk.pre~I(POD-0.5)*(",reg.dummies.frm,")+age+sex+MMSE+cc_score_pre +(1|subject_id)")) # as input for step()

md.pre.ext=lmer(formula.md.pre.ext,data=subset(md.data, !is.na(POD) & !is.na(age) & !is.na(sex) & !is.na(MMSE) & !is.na(cc_score_pre))) # creates extended mixed-effects models of preoperative data

fw.pre.ext=lmer(formula.fw.pre.ext,data=subset(fw.data, !is.na(POD) & !is.na(age) & !is.na(sex) & !is.na(MMSE) & !is.na(cc_score_pre))) # CAVE! rows with issing values have been removed beforehand

fa.pre.ext=lmer(formula.fa.pre.ext,data=subset(fa.data, !is.na(POD) & !is.na(age) & !is.na(sex) & !is.na(MMSE) & !is.na(cc_score_pre)))

mk.pre.ext=lmer(formula.mk.pre.ext,data=subset(mk.data, !is.na(POD) & !is.na(age) & !is.na(sex) & !is.na(MMSE) & !is.na(cc_score_pre)))

terms.md.pre.ext=(attr(terms(md.pre.ext),"term.labels"))[1:52] # defining a minimum set of variables to be

terms.fw.pre.ext=(attr(terms(fw.pre.ext),"term.labels"))[1:52] # included in the model (main effects only)

terms.fa.pre.ext=(attr(terms(fa.pre.ext),"term.labels"))[1:52] 

terms.mk.pre.ext=(attr(terms(mk.pre.ext),"term.labels"))[1:52]

stepw.reg.md.pre=step(object=md.pre.ext, alpha.fixed=0.05, reduce.fixed=TRUE, reduce.random=FALSE, keep=terms.md.pre.ext) # backward selection 

stepw.reg.fw.pre=step(object=fw.pre.ext, alpha.fixed=0.05, reduce.fixed=TRUE, reduce.random=FALSE, keep=terms.fw.pre.ext) # of significant POD 

stepw.reg.fa.pre=step(object=fa.pre.ext, alpha.fixed=0.05, reduce.fixed=TRUE, reduce.random=FALSE, keep=terms.fa.pre.ext) # by region interaction

stepw.reg.mk.pre=step(object=mk.pre.ext, alpha.fixed=0.05, reduce.fixed=TRUE, reduce.random=FALSE, keep=terms.mk.pre.ext) # terms

md.pre.red=get_model(stepw.reg.md.pre) # get the final models after backward

fw.pre.red=get_model(stepw.reg.fw.pre) # selection of significant interaction terms

fa.pre.red=get_model(stepw.reg.fa.pre)

mk.pre.red=get_model(stepw.reg.mk.pre)

#-----PRINTING REGRESSION ANALYSIS RESULTS (PREOPERATIVE DATA)------------

summary(md.pre.red)

summary(fw.pre.red)

summary(fa.pre.red)

summary(mk.pre.red)

#-----PREPARING REGRESSION MODELS FOR 3M-POSTOPERATIVE DATA ANALYSIS------

formula.md.3m.ext=as.formula(paste0("md.post~I(POD-0.5)*(",reg.dummies.frm,")+md.pre+age+sex+MMSE+cc_score_pre +(1|subject_id)")) # creates formulas

formula.fw.3m.ext=as.formula(paste0("fw.post~I(POD-0.5)*(",reg.dummies.frm,")+fw.pre+age+sex+MMSE+cc_score_pre +(1|subject_id)")) # of extended models

formula.fa.3m.ext=as.formula(paste0("fa.post~I(POD-0.5)*(",reg.dummies.frm,")+fa.pre+age+sex+MMSE+cc_score_pre +(1|subject_id)")) # of preoperative data

formula.mk.3m.ext=as.formula(paste0("mk.post~I(POD-0.5)*(",reg.dummies.frm,")+mk.pre+age+sex+MMSE+cc_score_pre +(1|subject_id)")) # as input for step()

md.3m.ext=lmer(formula.md.3m.ext,data=subset(md.data, !is.na(POD) & !is.na(age) & !is.na(sex) & !is.na(MMSE) & !is.na(cc_score_pre) & !is.na(md.pre))) # creates extended mixed-effects models of preoperative data

fw.3m.ext=lmer(formula.fw.3m.ext,data=subset(fw.data, !is.na(POD) & !is.na(age) & !is.na(sex) & !is.na(MMSE) & !is.na(cc_score_pre) & !is.na(fw.pre))) # CAVE! rows with missing values have been removed beforehand

fa.3m.ext=lmer(formula.fa.3m.ext,data=subset(fa.data, !is.na(POD) & !is.na(age) & !is.na(sex) & !is.na(MMSE) & !is.na(cc_score_pre) & !is.na(fa.pre)))

mk.3m.ext=lmer(formula.mk.3m.ext,data=subset(mk.data, !is.na(POD) & !is.na(age) & !is.na(sex) & !is.na(MMSE) & !is.na(cc_score_pre) & !is.na(mk.pre)))

terms.md.3m.ext=(attr(terms(md.3m.ext),"term.labels"))[1:53] # defining a minimum set of variables to be

terms.fw.3m.ext=(attr(terms(fw.3m.ext),"term.labels"))[1:53] # included in the model (main effects only)

terms.fa.3m.ext=(attr(terms(fa.3m.ext),"term.labels"))[1:53] 

terms.mk.3m.ext=(attr(terms(mk.3m.ext),"term.labels"))[1:53]

stepw.reg.md.3m=step(object=md.3m.ext, alpha.fixed=0.05, reduce.fixed=TRUE, reduce.random=FALSE, keep=terms.md.3m.ext) # backward selection 

stepw.reg.fw.3m=step(object=fw.3m.ext, alpha.fixed=0.05, reduce.fixed=TRUE, reduce.random=FALSE, keep=terms.fw.3m.ext) # of significant POD 

stepw.reg.fa.3m=step(object=fa.3m.ext, alpha.fixed=0.05, reduce.fixed=TRUE, reduce.random=FALSE, keep=terms.fa.3m.ext) # by region interaction

stepw.reg.mk.3m=step(object=mk.3m.ext, alpha.fixed=0.05, reduce.fixed=TRUE, reduce.random=FALSE, keep=terms.mk.3m.ext) # terms

md.3m.red=get_model(stepw.reg.md.3m) # get the final models after backward

fw.3m.red=get_model(stepw.reg.fw.3m) # selection of significant interaction terms

fa.3m.red=get_model(stepw.reg.fa.3m)

mk.3m.red=get_model(stepw.reg.mk.3m)

#-----PRINTING REGRESSION ANALYSIS RESULTS (POSTOPERATIVE DATA)-----------

summary(md.3m.red)

summary(fw.3m.red)

summary(fa.3m.red)

summary(mk.3m.red)

#-----PREPARING REGRESSION MODELS FOR 12M-POSTOPERATIVE DATA ANALYSIS-----

formula.md.12m.ext=as.formula(paste0("md.post_oneYearFU~I(POD-0.5)*(",reg.dummies.frm,")+md.pre+age+sex+MMSE+cc_score_pre +(1|subject_id)")) # creates formulas

formula.fw.12m.ext=as.formula(paste0("fw.post_oneYearFU~I(POD-0.5)*(",reg.dummies.frm,")+fw.pre+age+sex+MMSE+cc_score_pre +(1|subject_id)")) # of extended models

formula.fa.12m.ext=as.formula(paste0("fa.post_oneYearFU~I(POD-0.5)*(",reg.dummies.frm,")+fa.pre+age+sex+MMSE+cc_score_pre +(1|subject_id)")) # of preoperative data

formula.mk.12m.ext=as.formula(paste0("mk.post_oneYearFU~I(POD-0.5)*(",reg.dummies.frm,")+mk.pre+age+sex+MMSE+cc_score_pre +(1|subject_id)")) # as input for step()

md.12m.ext=lmer(formula.md.12m.ext,data=subset(md.data, !is.na(POD) & !is.na(age) & !is.na(sex) & !is.na(MMSE) & !is.na(cc_score_pre) & !is.na(md.pre))) # creates extended mixed-effects models of preoperative data

fw.12m.ext=lmer(formula.fw.12m.ext,data=subset(fw.data, !is.na(POD) & !is.na(age) & !is.na(sex) & !is.na(MMSE) & !is.na(cc_score_pre) & !is.na(fw.pre))) # CAVE! rows with missing values have been removed beforehand

fa.12m.ext=lmer(formula.fa.12m.ext,data=subset(fa.data, !is.na(POD) & !is.na(age) & !is.na(sex) & !is.na(MMSE) & !is.na(cc_score_pre) & !is.na(fa.pre)))

mk.12m.ext=lmer(formula.mk.12m.ext,data=subset(mk.data, !is.na(POD) & !is.na(age) & !is.na(sex) & !is.na(MMSE) & !is.na(cc_score_pre) & !is.na(mk.pre)))

terms.md.12m.ext=(attr(terms(md.12m.ext),"term.labels"))[1:53] # defining a minimum set of variables to be

terms.fw.12m.ext=(attr(terms(fw.12m.ext),"term.labels"))[1:53] # included in the model (main effects only)

terms.fa.12m.ext=(attr(terms(fa.12m.ext),"term.labels"))[1:53] 

terms.mk.12m.ext=(attr(terms(mk.12m.ext),"term.labels"))[1:53]

stepw.reg.md.12m=step(object=md.12m.ext, alpha.fixed=0.05, reduce.fixed=TRUE, reduce.random=FALSE, keep=terms.md.12m.ext) # backward selection 

stepw.reg.fw.12m=step(object=fw.12m.ext, alpha.fixed=0.05, reduce.fixed=TRUE, reduce.random=FALSE, keep=terms.fw.12m.ext) # of significant POD 

stepw.reg.fa.12m=step(object=fa.12m.ext, alpha.fixed=0.05, reduce.fixed=TRUE, reduce.random=FALSE, keep=terms.fa.12m.ext) # by region interaction

stepw.reg.mk.12m=step(object=mk.12m.ext, alpha.fixed=0.05, reduce.fixed=TRUE, reduce.random=FALSE, keep=terms.mk.12m.ext) # terms

md.12m.red=get_model(stepw.reg.md.12m) # get the final models after backward

fw.12m.red=get_model(stepw.reg.fw.12m) # selection of significant interaction terms

fa.12m.red=get_model(stepw.reg.fa.12m)

mk.12m.red=get_model(stepw.reg.mk.12m)

#-----PRINTING REGRESSION ANALYSIS RESULTS (POSTOPERATIVE DATA)-----------

summary(md.12m.red)

summary(fw.12m.red)

summary(fa.12m.red)

summary(mk.12m.red)
